# Using the exploration, preparation, implementation, sustainment (EPIS) framework to assess the cooperative re-engagement controlled trial (CoRECT)

**DOI:** 10.3389/fpubh.2023.1223149

**Published:** 2023-12-01

**Authors:** Heather Elder, Simona G. Lang, Merceditas Villanueva, Betsey John, Kathleen Roosevelt, Frederick L. Altice, Kathleen A. Brady, Briana Gibson, Marianne Buchelli, Alfred DeMaria, Liisa M. Randall

**Affiliations:** ^1^Massachusetts Department of Public Health, Boston, MA, United States; ^2^Yale University School of Medicine, New Haven, CT, United States; ^3^Philadelphia Department of Public Health, Philadelphia, PA, United States; ^4^Connecticut Department of Public Health, Hartford, CT, United States

**Keywords:** HIV, implementation science, out-of-care, re-engagement, RCT – randomized controlled trial, EPIS framework

## Abstract

**Background:**

“Data to Care” (D2C) is a strategy which relies on a combination of public health surveillance data supplemented by clinic data to support continuity of HIV care. The Cooperative Re-Engagement Controlled Trial (CoRECT) was a CDC-sponsored randomized controlled trial of a D2C model, which provided an opportunity to examine the process of implementing an intervention for people with HIV (PWH) who are out-of-care across three public health department jurisdictions. Using the EPIS (Exploration, Preparation, Implementation, Sustainment) framework, we aimed to retrospectively describe the implementation process for each site to provide insights and guidance to inform future D2C activities implemented by public health agencies and their clinical and community partners.

**Methods:**

After completion of CoRECT, the three (Connecticut, Massachusetts, Philadelphia) trial sites reviewed study protocols and held iterative discussions to describe and compare their processes regarding case identification, interactions with partnering clinics and patients, and sustainability. The EPIS framework provided a structure for comparing key organizational and operational practices and was applied to the entire implementation process.

**Results:**

The trial sites varied in their implementation processes and the specific elements of the intervention. Factors including prior D2C experience, data management and analytic infrastructure, staff capacity, and relationships with clinic partners informed intervention development and implementation. Additionally, this review identified key lessons learned including to: (1) explore new supplemental sources for public health surveillance data; (2) work with stakeholders representing core functions/components in the early stages of the intervention design process; (3) build flexibility into all components of the follow-up activities; and (4) integrate data sharing, project management, and follow-up activities within existing DPH organizational structure.

**Conclusion:**

The CoRECT study provides a general blueprint and lessons learned for implementing a D2C intervention for re-engagement in HIV care. Interventions should be tailored to local operational and structural factors, and responsive to evolving clinical and public health practices.

## Introduction

The National HIV/AIDS Strategy, the United States plan for addressing and ending the HIV epidemic, prioritizes increasing rapid linkage to care and improving retention in care to achieve viral suppression (1, 2). The HIV care continuum is a conceptual public health tool used by the Centers for Disease Control and Prevention (CDC) to monitor progress toward reaching national goals and is comprised of the diagnosis, linkage, and retention steps necessary to achieve viral suppression. Identified implementation gaps guide potential opportunities to improve engagement in care (3).

Various models of care have been developed to improve retention in care and viral suppression among people with HIV infection (PWH). An initial step in these care models is to define PWH who are out-of-care (OOC), which can vary (4). The CDC, the Health Resources and Services Administration (HRSA), and the Institute of Medicine (IOM, now National Academy of Medicine) offer differing definitions of retention in HIV care (1, 5–8). For example, the CDC defines retention in care as having at least two CD4 cell counts or viral load tests performed at least 3 months apart within 1 year, while the IOM and HRSA define retention as at least two medical visits every 12 months, with a minimum of 90 days between visits (5, 8).

Different clinic-based and public health strategies have been used to improve retention in HIV care. “Data to Care” (D2C), is one strategy that uses a combination of public health surveillance and other, often clinical, data to increase the number of PWH receiving sustained HIV care and viral suppression (9, 10). The CDC developed a toolkit for designing D2C interventions in 2017. Implementation across diverse health department jurisdictions, however, varies based on available data, methods for data collection, data management systems and processes, data sharing policies, and health department capacity, funding, and infrastructure (4).

The Cooperative Re-Engagement Controlled Trial (CoRECT) was a CDC-sponsored randomized control trial (RCT) of a D2C model with a research goal to establish data-sharing partnerships between health departments and HIV care clinical providers designed to identify and re-engage PWH that are OOC. This goal was operationalized by: (1) identifying newly OOC PWH through a collaborative data-sharing approach using public health surveillance and clinical data; and (2) implementing a public health outreach intervention to improve HIV care re-engagement, retention in care, and viral suppression for OOC PWH. Each of these elements differed by site. All three sites in this trial (Connecticut, Massachusetts, and Philadelphia health departments), found that a collaborative D2C strategy was successful at re-engaging PWH into HIV care at 90 days compared to the standard of care (11). Only the Philadelphia site resulted in downstream benefits of significantly improved retention in care at 12 months and viral suppression (10).

CoRECT’s D2C approach varied among the three participating health department jurisdictions and therefore provides fertile ground for compiling experiences and insights across multiple contexts. The objective of this study was to use a standardized implementation framework, EPIS (Exploration, Preparation, Implementation, Sustainment), to describe the implementation process for each of the three CoRECT sites as part of a retrospective assessment in order to better understand and appraise the components of the D2C intervention. Defining, clarifying, and analyzing D2C implementation within the EPIS framework enabled us to articulate lessons learned that can inform delivery, sustainability and replicability of future D2C activities.

## Methods

### Overview of the CDC’s cooperative re-engagement controlled trial

CoRECT was a multisite, randomized controlled trial to implement and evaluate a public health intervention compared to clinic standard of care (SOC) for re-engaging OOC PWH, using D2C. Participants were recruited from 8/2016–7/2018. The *a priori* definition for OOC included receiving HIV care at a participating clinic for at least 12 months and then subsequently neither a visit with a prescribing provider nor CD4 or viral load test result reported to health department surveillance for at least 6 months. Eligible OOC patients were randomized within each clinic to the clinic SOC for patient follow-up, or a D2C health department intervention in which patient follow-up was performed by public health field epidemiologists/Disease Intervention Specialists (DIS). There was a basic structure to the trial across all sites and participating clinics, but specific intervention characteristics varied across the three sites, with adjustments at each clinic consistent with variations in workflow, staffing, and capacity. Each site used a “case conferencing” methodology where health department staff interacted with clinic staff to reach consensus on persons who were OOC and eligible for randomization. After case randomization, public health field staff undertook patient outreach activities culminating in a warm handoff to clinic staff. The health department project management teams monitored surveillance data to determine re-engagement, viral suppression, and retention outcomes.

The Connecticut Department of Public Health/Yale School of Medicine (CTDPH/YSM), the Massachusetts Department of Public Health (MDPH), and the Philadelphia Department of Public Health (PDPH) participated as the three CoRECT sites. In 2016, Connecticut, Massachusetts, and Philadelphia reported approximately 10,286 (335 per 100,000), 20,060 (342 per 100,000), and 19,113 (1,252 per 100,000) PWH residing in their jurisdictions, respectively (12, 13). These sites estimated about 24 to 54% of their PWH met the OOC definition.

**Figure 1:**
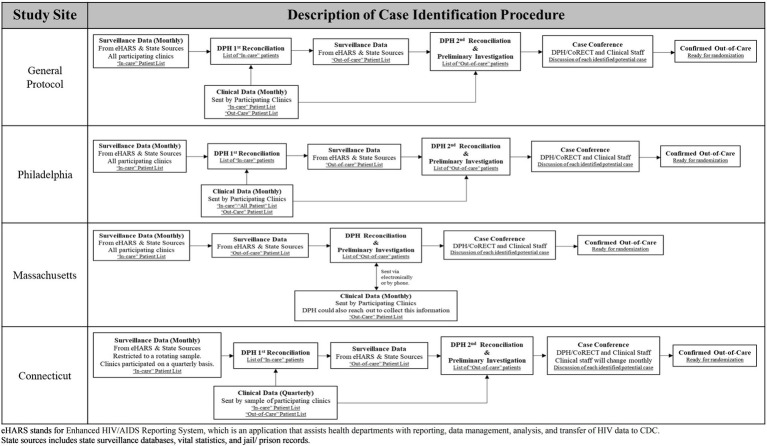
Description of case identification procedures used by each CoRECT site.

The CoRECT intervention allowed for site-specific flexibility regarding the case identification process and the re-engagement follow-up activities. Figure 1 illustrates how each site adapted the standard definition for case identification based on variations in workflow, data infrastructure, and communication processes at the sites and participating clinics. Table 1 provides further details regarding differences by site in the areas of case identification, case conference characteristics, randomization, and re-engagement follow-up activities, including field epidemiologist/DIS characteristics and contact methods, and intervention characteristics.

**Table 1:** Organizational and operational aspects of CoRECT in the participating jurisdictions.

	Connecticut/Yale	Massachusetts	Philadelphia
Study population and patient identification			
Participating clinics	23 clinics in 4 counties.	9 clinics in 4 counties.	8 clinics in one large city.
Clinic settings/types	Ryan White-funded; community health centers; hospital-based and private clinics.	Community health centers; not-for-profit, non-academic hospitals; a public health hospital; and non-profit, academically-affiliated teaching hospitals.	Ryan White funded; FQHC; academic institutions; a Veterans’ Administration medical center; private clinic.
OOC patient population estimate	>7,500 patients, of which 1,700 were estimated to be out-of-care.	>6,000 patients, of which 1,300 were estimated to be out-of-care.	4,793 patients, of which 1,367 were estimated to be out-of-care.
OOC patient population percent	33%	24%	54%
Used pilot period	No	No	Yes
DPH patient identification	in-care: Monthly, a statewide list was generated from eHARS. Restricted to patients at participating clinics. out-of-care: Monthly, a list from eHARS was generated, stratified by CoRECT clinic, starting from the initial “in care” list.	in-care: No separate lists were generated but included as a part of the out-of-care list generating algorithm. out-of-care: Monthly, a list was generated from surveillance data for participating clinics/departments within participating facilities.	in-care: No list. out-of-care: Monthly, a list was generated starting from the initial clinic provided “in care/all patient” list.
Clinic patient identification	in-care: Quarterly, list of patients using clinical and/or appointment records. out-of-care: Quarterly, list of patients suspected to be potentially out-of-care using clinical and/or appointment records.	“in-care”: No list. “out-of-care”(OOC)/“all patient”: Monthly, clinics had three paths for providing patient information. 1. Generate OOC patient list using clinical and/or appointment records. 2. Generate “all patient” list with additional variables to help determine their OOC status. 3. Clinics with small numbers of patients could opt to provide their OOC patients verbally.	in-care/all patient: Monthly, list of patients using clinical and/or appointment records. out-of-care: No list.
List reconciliation	DPH reconciled initial OOC list with that provided by participating clinics before searching other databases. Patients were classified based on clinic visit attendance and VL data. These lists were returned to clinics for case conferences.	DPH reconciled initial OOC list with those provided by participating clinics before searching other databases. Only cases identified as OOC by both DPH and participating clinics were assessed in the preliminary adjudication.	DPH reconciled initial OOC list with all patient list provided by the participating clinics before searching other databases.
Method for data exchange.	Electronically via secure file transfer	Electronically or by phone	Electronically
Case conference characteristics			
Location of conference	In-person and phone	In-person and phone	In-person (majority) and phone
Frequency of clinic participation	Quarterly for each clinic	Monthly for each clinic	Monthly for each clinic
Estimated average conference length	Up to 1 h	Approximately 1 h	1 to 2 h
DPH/CoRECT staff descriptions	CT DPH HIV Epidemiologist: Participated as needed; kept notes; recorded final case disposition; and sent final list of out-of-care patients for randomization. Yale Data Manager: Streamlined patient discussion and assist with finalizing list for randomization. DIS: Participated as needed.	MDPH Data to Care Epidemiologist: Led conference call; kept notes; recorded final case disposition; and conducted the randomization after the conference.	PDPH Data to Care Project Coordinator: Led conferences; kept notes; recorded final case disposition; and sent final list of out-of-care patients for randomization. Assisted by PDPH Data to Care Data Managers and/or Epidemiologist.
Clinic staff participant descriptions	Not all participated in each conference call: • Case managers • Social workers • Clinicians • RNs	The primary clinic representative and other potential attendees included: • Case managers • Data managers • Social workers, patient navigators • Clinicians • Nursing staff	Not all participated in each conference call: • Case managers • Social workers • Clinicians • RNs
Structure of conferences	CT DPH staff or Yale Data Manager reviewed the list of potential cases with clinic staff (did not specifically go through each case) and answered disposition questions; ensured clinics were confident in patients designated to be randomized. There was no consistent structure to the conferences over the course of the study period. Conferences generally happened by phone, occasionally face to face. Clinics were also given option to provide case dispositions via spreadsheet.	MDPH called clinic with a list of patients most likely to be OOC. During the reconciliation call patients are removed from the list if determined to be in care or clinician discretion. The remaining patients on the list were then discussed in more detail to confirm and/or obtain key information necessary for field follow-up, including demographics and locating information.	The PDPH Data to Care Project Coordinator reviewed each case on the list of potentially OOC with clinic staff and answered disposition questions; ensured clinics were confident in patients’ eligibility to be randomized. Conferences mainly happened in-person, but occasionally by phone if case conference list was less than 5 cases.
Case conference characteristics			
Patients discussed during conference	All patients who appear to have disengaged from care after the DPH preliminary investigation. Some potentially eligible patients were put on a watch list for further review. Each clinic did their preliminary reconciliation to exclude patients not eligible for randomization.	All patients who appear to have disengaged from care after the DPH preliminary adjudication. Some potentially eligible patients were put on a watch list for further review based on provider feedback.	All patients who appear to have disengaged from care after the DPH preliminary investigation. Some potentially eligible patients were put on a watch list for further review based on provider feedback.
Randomization			
Randomization method	Permuted block randomization by clinic. Randomization occurred within 10 days of the case conference. Clinic staff were blinded to the randomization assignment. Randomization was done by Yale School of Medicine research staff through the REDCap data management system. The randomized individuals were communicated back to the DPH where they were assigned to DIS. Patients could only be randomized once.	Permuted block randomization by clinic. Randomization occurred within 2 days of the case conference. Clinic staff were blinded to the randomization assignment. Randomization was done by MDPH surveillance staff using SAS 9.3. Patients could only be randomized once.	Permuted block randomization by clinic. Randomization occurred within 5 days of the case conference. Clinic staff were blinded to the randomization assignment. Randomization was done by the CoRECT data manager using SAS. The patients randomized to the intervention were sent to the STD Control program in the Division of Disease Control for assignment to the PDPH outreach staff. Patients could only be randomized once.
Field staff/discharacteristics			
Number of field staff	3	8–10	2–3
Level of staff	Local DIS that were hired through local health departments; these were new DIS as opposed to reassigned traditional DIS.	State field epidemiologists conducting HIV/STI follow-up activities.	Local DIS were hired. The DIS who took the positions were transferred from traditional DIS roles.
Other required duties	Dedicated to this study however were given additional roles such as working with new HIV diagnoses as needed.	1. Active patient follow-up for infectious syphilis, including HIV co-infected cases, and acute HIV cases. 2. Provider requested HIV follow-up. 3. Follow-up for other supplemental projects. 4. Active follow-up for HIV clusters. 5. Follow-up on untreated female chlamydia cases of childbearing age. 6. Provider and patient follow-up with rectal gonorrhea cases and untreated gonorrhea cases.	1. Covered public health clinics. 2. When required, took on additional syphilis and HIV case assignments.
Field staff/DIS characteristics			
Training	Standard DIS training and an adapted “Anti-Retroviral Treatment and Access to Services” (ARTAS) intervention.	Standard DIS training, training on motivational interviewing, and the CoRECT intervention protocol.	Standard DIS training, re-engagement training based on local protocol, and an adapted ARTAS intervention.
Status of Out-posting	DIS were stationed at local health departments in 3 metropolitan areas.	Field epidemiologists were located at MDPH, regional MDPH offices, and several were outposted/embedded part-time at collaborating clinics.	DIS were located at PDPH.
Contact methods and intervention characteristics			
Locating and contact methods	Lasted up to 30 days after randomization. Utilized Lexis Nexis, clinical based information, and surveillance data. Attempts were made via phone calls, social media, certified mail, field visits to home, work, friends and next of kin.	Lasted up to 30 days after randomization. Used contact and emergency contact information reported by the last documented clinical provider and conducted public records review. Attempts were made via phone, text messaging, mailed letters, social media, email, and field visits.	Lasted up to 90 days after randomization. Utilized Lexis Nexis, clinical based information, and surveillance data. Attempts were made via phone calls, mail, social media, and field visits to home.
Intervention components	An adapted ARTAS intervention. Prior to 1^st^ clinic visit, DIS worked with patients to assess the readiness to re-engage into care as well as determine the need for additional interventions. DIS worked with clinics to schedule appointments within 24 h of patient contact (if possible). 1^st^ visit (within 6 weeks): If needed, DIS escorted patients to the visit. Additionally, DIS administered a brief barrier to care survey based on the mHealth Survey. Based on the patient assessments, DIS and clinical staff were equipped to provide other community resources. 2^nd^ visit (within an additional 6 weeks): If needed, DIS assisted with scheduling this appointment.	Once contact with patients was established, field epidemiologists attempted to initiate engagement in care. Outreach continued until patient either agreed to reengagement assistance, declined assistance, or was lost-to-contact after a 60-day period following initial contact. Initial interview: Field epidemiologists assessed barriers and facilitators to care engagement via standardized interview guide and provided basic education relevant to needs of patient. Field epidemiologists worked with patients to develop a care engagement plan. This included identifying immediate practical support required to facilitate initial clinical visit, critical supports required to facilitate initial care engagement, and others related to long-term care retention. Field epidemiologists worked with clinics to expedite appointments within 24 h, if possible.	An ARTAS intervention: Five modules were offered over a 90 day period or up to five visits (home or other location) whichever happened first. Major ARTAS module components: *1. Building the Relationship* *2. Barriers Assessment* *3. Patient Education* *4. Strengths Assessment* *5. Goal Setting* *6. Identifying and Linking to Community Resources* *7. Linkage to Care* Initial interview: Patients were given information about the importance of connecting with medical care. DIS assessed barriers and facilitators to care engagement to help gauge future goal setting, strength building and resource referral services. DIS shared barrier information with clinical providers with patient consent.
Contact methods and intervention characteristics			
Intervention components (continued)	Engagement verification: To assure that patients re-engaged as in kept clinic visit, a Yale research staff associate contacted the clinics to verify appointment.	Information was provided to the clinic about barriers and facilitators to care engagement/retention, referrals made for social services and other services, and plans for longer-term engagement assistance and support. If needed, field epidemiologists could provide patients with appointment reminders and accompany patients or coordinate transportation to/from clinical visits and other support services. Engagement verification: To assure initial reengagement and facilitate retention in care, field epidemiologists followed-up with clinics to verify engagement within three days of the scheduled appointment. If the patient did not attend appointment, then additional attempts to re-contact/re-interview were made in a renewed period of 30 days following the missed appointment.	Distribution of modules over visits: The first three patient contacts consisted of relationship building, identifying strengths, addressing patient needs and care barriers, and encouraging linkage to care. If needed, two more interaction were permitted. DIS only presented applicable components or modules. DIS worked with clinics to expediate appointments within 24 h, if possible. Transition to Care Phase: After the initial clinical visit, DIS were able to continue patient follow-up for 60 days to provide any additional support. Ascertaining labs: Once patients had a verified visit, DIS followed the patient in the surveillance system for up to 60 days to determine if labs were completed.
Long-term patient follow-up	At any time during the intervention, DIS were able to introduce patients to an Early Intervention Specialist (EIS). When invoked, EIS assisted clients with multiple unmet needs (e.g., addiction treatment, mental health services, housing assistance, etc.). Additionally, EIS provided a more intensive linkage to Ryan White-funded services and were not limited by DIS time frames. In general, MCM services when available (specifically in Ryan White-funded sites) were charged with retention approaches together with the clinic’s usual standard of care.	The protocol allowed field epidemiologists to provide patients with longer-term support and assistance for up to one year including accompanying patients to/from clinical visits and other prevention or support services, providing prevention counseling to support ongoing risk reduction, and identifying and engaging with alternative care providers.	DIS had the ability to use a comprehensive HIV Medical Case Management (MCM) network that is available to provide long term intensive services, (including appropriate referrals), to patients that may exhibit barriers that cannot be resolved within the brief intervention. All patients already enrolled in MCM were offered the service and these patients received priority statis for MCM services by MCM central intake.

### The exploration, preparation, implementation, and sustainment framework

While the EPIS framework was originally developed to aid in the development, execution, and sustainment of research interventions in a prospective manner, we chose to use this framework retrospectively to review implementation outcomes, which has been done previously elsewhere. Our rational for including implementation outcomes is based on a systematic review that suggests that most RCTs fail to report implementation outcomes, including retrospectively (14, 15). The four distinct phases – exploration, preparation, implementation, and sustainment – represent stages in the development and execution of a research intervention (16, 17). Each phase can be parsed into the outer context (e.g., the service and policy environment, target population characteristics, and inter-organizational relationships), inner context (e.g., leadership, organizational structures and resources, and staffing, of the implementing organization which can vary), innovation (e.g., how it fits into the organization, provider, and patient levels), and bridging factors (e.g., community-academic partnerships). Multiple factors comprise each of the contextual levels. The factors that comprise the outer context include sociopolitical, funding, interorganizational networks, and leadership, while organizational characteristics, individual adopter characteristics, leadership, participant recruitment, fidelity monitoring/support, and staffing make up the inner context. Both innovation and bridging factors are unifactorial; innovation characteristics and community and academic partnerships, respectively (14, 16). Reviewing the factors during each phase allows for the identification of barriers and facilitators to implementation.

### Retrospective assessment of CoRECT implementation

To understand the different outcomes at each site, the three jurisdictions decided to evaluate implementation and associated processes to inform continued and future interventions, and identify key lessons that could assist further implementation of D2C programs. The process consisted of reviewing study protocols and holding discussions among primary investigators and project team members from each site. Using the EPIS framework, sites described and documented their processes for case identification, collaboration, and communication with partnering clinics, and interactions with patients (see Table 1 for details). The EPIS framework was selected because of its utility in understanding implementation within real-world settings and allowance for the systematic comparison of the trial sites according to key organizational and operational practices, enabling analysis of future delivery and sustainability of each CoRECT intervention component.

## Results

### Organizational and operational characteristics of DPH sites

The three trial sites differed in their implementation of CoRECT study activities. CTDPH/YSM recruited 23 clinics across four counties (out of eight counties statewide) and included community health centers, hospital-based, and other clinics funded by federal Ryan White HIV/AIDS Program. MDPH recruited nine clinics across four counties (out of 14 counties statewide) and included community health centers, a hospital-based practice, a public health hospital, and non-profit, academically affiliated teaching hospitals. Participating clinics represented a mix of funding sources (including Ryan White) and included facilities that received no dedicated HIV services funding. PDPH recruited eight clinical sites all within the City of Philadelphia. These participating clinics included Ryan White-funded community health centers and academic medical centers, as well as a Veterans Health Administration medical center and a private clinic (Table 1).

Table 1 provides a detailed description of each of the 3 sites organized by study population, case conferencing methodology, randomization strategy, field epidemiologist/DIS characteristics, contact methods and intervention characteristics.

### Analysis of the CoRECT intervention elements using EPIS framework

**Figure 2:**
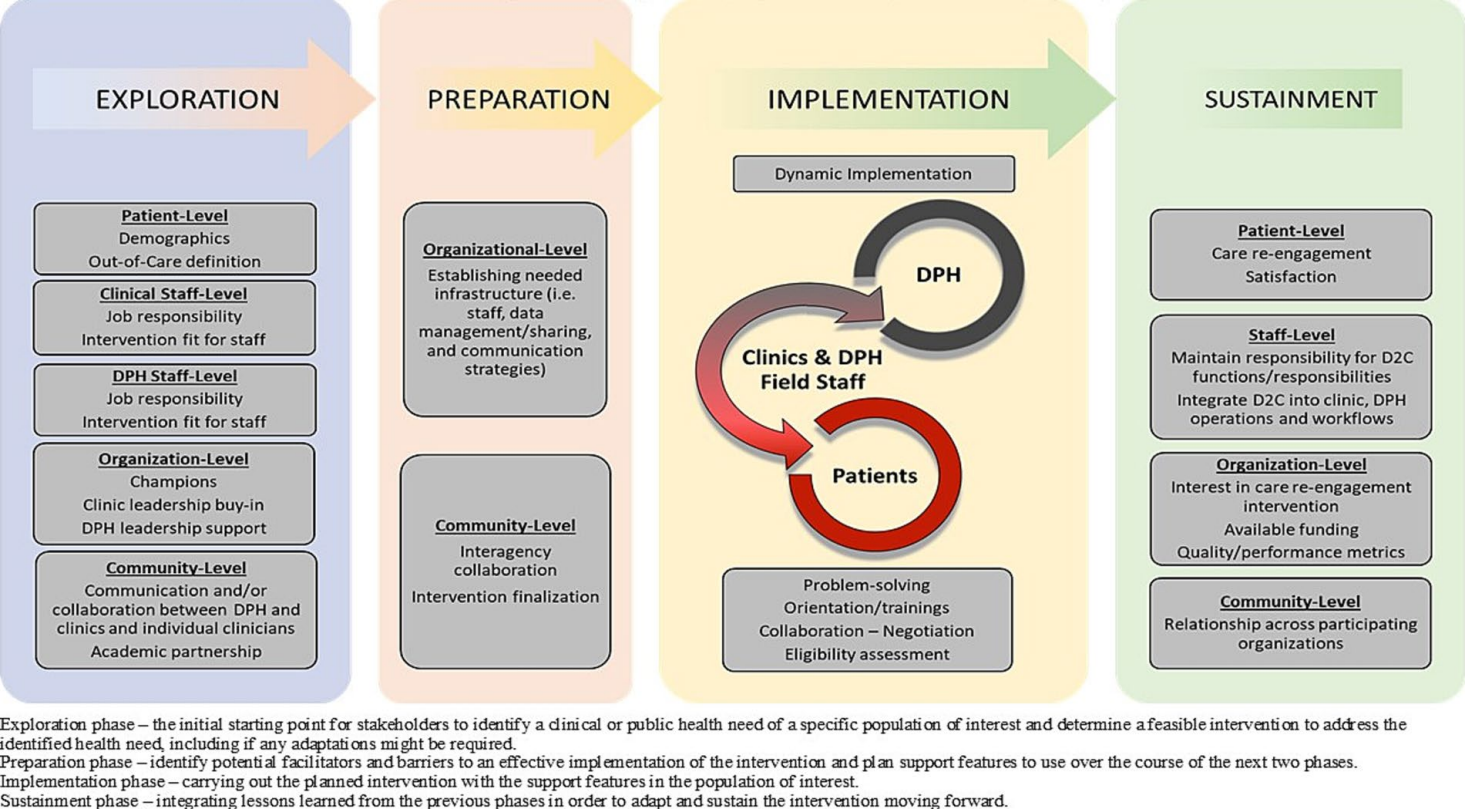
CoRECTs implementation within the Exploration, Preparation, Implementation, Sustainment (EPIS) framework.

Figure 2 illustrates how implementation of CoRECT fits within the four phases of the framework. The following highlights key selected findings within each of the 4 phases: Exploration, Preparation, Implementation and Sustainment.

### Exploration phase

#### Interorganizational networks

Strengthening collaboration between health departments and healthcare clinics was an explicit, secondary goal of CoRECT. Through strengthened collaboration, CoRECT provided sites with new tools to assist patients and healthcare providers with re-establishing engagement in HIV care (Supplementary Table 1).

#### Leadership

The leadership across the three participating DPHs prioritized applying data to public health action, developing/adopting methods for promoting and supporting quality improvement, promoting equity in access to healthcare, and achieving health outcomes. Additionally, primary consideration was given to addressing known gaps in HIV care including using novel D2C methods for improving care engagement.

#### Organizational characteristics

CTDPH, PDPH, and MDPH had varying levels of experience with D2C activities and participation in other HIV care research studies. Although CTDPH had no prior D2C experience, they relied on experienced HIV surveillance staff and prior experience working with Yale researchers on other related projects. PDPH staff had some prior D2C experience, in addition to the pilot, which preceded their CoRECT enrollment to test patient identification procedures. Prior to CoRECT, PDPH had received Gilead funding for START Care, which assessed data exchanges with providers to determine OOC patients. Unlike CoRECT, providers were responsible for directly administering the intervention. MDPH had prior D2C experience through SPECTRuM and Partnerships for Care (18–20).

#### Staffing

The three sites used their existing field investigation/patient follow-up activities as a base for implementing their public health field intervention. MDPH used existing field staff and integrated OOC engagement and navigation activities into existing functions associated with public health follow-up of syphilis and acute HIV, while PDPH and CTDPH/YSM hired designated CoRECT field staff focused primarily on re-engagement of OOC clients and assisted with other outreach activities as needed. The field staff (DIS) in Connecticut were hired and housed by local health departments (Bridgeport, New Haven, Hartford). PDPH hired and managed designated CoRECT field staff through STD Control Program within the Division of Disease Control. CTDPH/YSM coordinated their CoRECT outreach activities based on a combination of local and state HD practices. All three sites provided training on study procedures to field staff; training of clinic staff regarding study goals and procedures varied at participating clinics.

Data management staff varied among the sites. For MDPH, CoRECT data management activities were integrated with routine HIV/STD surveillance activities and coordinated by dedicated D2C staff. PDPH relied on a D2C data manager to work closely with a D2C project coordinator, both within the HIV Surveillance Unit, which would coordinate intervention follow-up activities with STD Control Program. Unlike the previous sites, CTDPH relied on their collaborators at YSM to perform data management including randomization and communication with local HDs.

#### Innovation characteristics

Sites had the flexibility to tailor both of the main study components (i.e., the patient identification process and patient follow-up). For example, sites used the initial general patient identification protocol (Figure 1) as a basis for discussion with partnering clinics about how to adjust the protocol to fit their individual operational and structural factors and abilities.

For follow-up of OOC patients by DIS, sites designed feasible public health outreach and patient navigation activities which encompassed short-term re-engagement assistance (similar to traditional public health disease intervention and follow-up) and longer-term case management (similar to traditional HIV medical case management or HIV prevention case management). CTDPH/YSM (Yale School of Medicine) implemented short-term patient re-engagement assistance through the use of a modified Anti-Retroviral Treatment and Access to Services (ARTAS) (21, 22) intervention. MDPH implemented a middle range approach by having a focus on short-term assistance with the possibility of longer-term assistance and motivational counseling for patients who needed or requested it, an adaptation of their standard procedures for persons with newly diagnosed HIV. MDPH further tailored patient follow-up activities to minimize collisions with ongoing HIV care initiatives. PDPH approach prioritized longer-term patient re-engagement assistance based on identified barriers to care and they modified their ARTAS approach by not including incentives (Table 1).

#### Community and academic partnerships

CTDPH supplemented their in-house knowledge and skills by partnering with YSM, leveraging their history of collaborating on grant-funded projects. YSM provided CTDPH with additional capacity to manage and execute the CoRECT trial. MDPH and PDPH relied on and benefitted substantially from prior experience and partnership with the participating clinics to manage and execute the intervention. Many of their clinic partners had experience in participating in HIV-related research, both clinical and interventional. Additionally, PDPH partnered with the University of Pennsylvania CFAR to serve as the community advisory board for the CoRECT project.

### Preparation phase

The trial sites collaborated closely with participating clinical partners to tailor the patient identification protocols (Figure 1) and provide relevant staff trainings (Supplementary Table 2).

#### Interorganizational networks

All the trial sites highlighted the importance of establishing a strong buy-in internally among DPH staff and administration as well as the staff and administration of participating clinics with which the CoRECT teams would be working closely. Cultivating relationships with the clinics consisted of explaining the project’s importance, the benefits to patients and clinical practice, and clear expectations (submitting patient lists, discussion of individual cases at conferences, and expedited appointments for randomized patients). Seeking buy-in of DPH staff differed based on their roles within the CoRECT team and other duties. Trial sites and their collaborating partners also established data sharing agreements.

#### Individual adopter characteristics

Both MDPH and PDPH relied on internal staff to coordinate project activities. This approach increased the sense of DPH “project ownership” which improved staff buy-in. For CTDPH/YSM, YSM staff served as project coordinator. Use of an external (third-party) coordinator in which clinic partners and CoRECT-specific DIS staff did not interact directly with CTDPH staff impacted staff “buy-in.” All three sites identified having a dedicated project contact within each collaborating clinic as critical for streamlining communication and improving implementation of patient follow-up activities.

PDPH differed from the other sites in that they conducted a pilot study to assess procedures and collect feedback from clinic leadership and staff prior to the implementation of the full trial. Based on the findings from the pilot, PDPH CoRECT team modified the initial patient identification protocol to the one shown in Figure 1 and determined the best format for case conferences.

#### Participant recruitment

The first stage of patient recruitment was case identification which had two components: data sharing/reconciliation and case conferences. Each trial site had a general protocol which could be adapted during the implementation phase. Figure 1 illustrates how the trial sites adapted a general protocol to meet their needs and those of their clinical partners. Case conferences were the principal forum for health department and clinic staff to discuss and review patient eligibility for the trial. They could be conducted in-person or by phone and occurred monthly or quarterly. Table 1 provides a detailed comparison of conference characteristics across the three trial sites.

Through their pilot study, PDPH determined that it was most productive to have their clinic partners submit “in-care” or “all patients” lists and to have structured in-person case conferences led by the PDPH project coordinator.

#### Staffing

The trial sites used this phase to onboard and train staff. Table 1 provides details regarding the qualifications of field staff. Non-field project staff were trained to perform case identification through surveillance and clinic data exchange and reconciliation, participate or lead case conferences, and randomization of cases. At CTDPH/YSM, the CT DPH HIV epidemiologist and the data manager located at YSM received these trainings. At PDPH, they trained the D2C project coordinator, the D2C managers, and D2C epidemiologists. In contrast, MDPH trained their D2C epidemiologist to manage all these tasks with some assistance provided by other HIV/STD surveillance epidemiologists.

#### Innovation characteristics

The ability of the CoRECT sites to adapt/modify general case identification protocols and patient follow-up activities were key innovative characteristics. DPH and YSM CoRECT teams worked closely with clinic partners to develop novel data sharing and case review protocols that were feasible for all parties and allowed for the timely identification of OOC cases.

### Implementation phase

The implementation phase of active recruitment highlighted the need to address unforeseen challenges during the RCT or for later consideration in the sustainment phase (Table 2).

**Table 2:** Implementation phase CoRECT project.

	EPIS constructs	Connecticut DPH	Massachusetts DPH	Philadephia DPH
**Outer context**	Funding	- Local health departments were contracted to hire CoRECT specific DIS.	- Clinics were funded to compensate for time and effort of staff for project-related activities including case conferences.	
	Interorganizational Networks	- Worked to strengthen relationships with participating clinics throughout the intervention.	- Worked to strengthen relationships with participating clinics throughout the intervention. - Clinics and DPH had prior experience collaborating on HIV-related programs, and prior D2C activities. - DPH supported additional educational and joint programs with providers and community organizations.	- Worked to strengthen relationships with participating clinics throughout the intervention. - Clinics and DPH had prior experience collaborating on HIV-related programs, and prior D2C activities.
	Leadership	- Supported and prioritized infrastructure development and expanding the capacity for data sharing and staff training.	- Supported and prioritized infrastructure development and expanding the capacity for data sharing and staff training.	- Supported and prioritized infrastructure development and expanding the capacity for data sharing and staff training.
**Inner context**	Organizational characteristics	- Yale School of Medicine (YSM) data manager randomized patients and worked with clinics to promote patient list generation and informal trainings. - DIS stationed at local health departments.	- Generated out-of-care lists and randomized patients. - Field staff integrated HIV out-of-care engagement patients into their work stream.	- Randomized patients by PDPH staff. - Hired local DIS to work on intervention and assist with additional case work.
	Individual adopter characteristics	- DIS were engaged and motivated to assist patients. - Providers were invested in getting patients re-engaged into care, but buy-in varied across clinics and impacted list generation and care re-engagement.	- Field staff brought specialized skills, nimbleness, and were very engaged and motivated to aid patients. - Clinic providers were very invested in getting patients re-engaged into care.	- DIS were engaged and motivated to assist patients. - Clinic providers were very invested in getting patients re-engaged into care.
	Leadership	- Competing priorities made it difficult to maintain full engagement. - YSM did not have oversight over DIS and clinic personnel.	- Competing priorities made it difficult to maintain full engagement.	- Remained engaged during the project.
	Participant recruitment	- Quarterly electronic data transmission by clinics. - Case conferences structure varied by clinics and staff availability. - Case conferences run by YSM data manager.	- Adapted case conference structure to better suit clinical needs. - Case conferences run by D2C epidemiologist. - Provided clinics with three methods for reporting either “all patient” or OOC lists.	- Adapted case conference structure to better suit clinical needs. - Case conferences run by D2C project coordinator. - Initially clinics submitted “in-care” lists but were subsequently allowed to submit “all patient” lists. - Case conferences were held in person.
**Inner context**	Fidelity monitoring/support	- Worked with YSM and clinics to ensure needed support was provided. - Held quarterly case conferences and periodic project status meetings with clinics to identify challenges. - Monthly internal meetings to address challenges. - No regular monitoring meetings with local health departments and YSM.	- Held monthly case conferences and periodic project status meetings with clinics to identify and address implementation challenges. - Monthly internal meetings to address challenges. - Routine review/Continuous Quality Improvement (CQI) of field staff activities associated with care re-engagement.	- Worked with participating clinics to ensure needed support was provided. - Held monthly case conferences and periodic project status meetings with clinics to identify challenges. - Monthly internal meetings to address challenges. - Routine review/CQI of field staff activities associated with care re-engagement.
	Staffing	- DIS hired through local health departments. - Existing clinic staff participated in out-of-care engagement activities by integrating it into workflows. - Needed to train extant and any new staff brought on during the implementation of the intervention. - Extant DIS were not integrated into the intervention.	- Out of care re-engagement functions were integrated into the scope of work of field epidemiologists. - Existing clinic staff participated in out-of-care engagement activities by integrating it into workflows. - Other demands competed with CoRECT activities. - Needed to train extant and any new staff brought on during the implementation of the intervention.	- Hired local DIS, data management and epidemiology staff for this intervention. - Existing clinic staff participated in out-of-care engagement activities by integrating it into workflows. - Needed to train extant and any new staff brought on during the implementation of the intervention.
**Innovation**	Innovation characteristics	- DIS provided additional assistance to patients and completed a modified ARTAS intervention. - DIS worked directly with clinics to schedule appointments quickly. - Quarterly conference calls. - Clinics generated out-of-care lists using clinic/appointment data; used combined clinic-surveillance data for out-of-care follow-up. - Clinics accepted warm hand-offs, received supplemental barrier to care information, and care reengagement support.	- Identified out-of-care HIV patients through data sharing with clinics. - Clinics generated of out-of-care lists using clinic/appointment data; used combined clinic-surveillance data for out-of-care follow-up. - Field epidemiologists facilitated “warm hand-offs” of patients to clinics, and additionally provided clinic staff with supplemental barrier to care information. - Field staff worked directly with clinics to schedule expedited appointments (i.e., within 24 h of patient interview). - Monthly conference calls.	- DIS provided additional assistance to patients and completed a non-incentive based ARTAS intervention. - DIS worked directly with clinics to schedule appointments quickly. - Monthly case conferences. - Clinics generated of out-of-care lists using clinic/appointment data; used combined clinic-surveillance data for out-of-care follow-up. - Clinics accepted warm hand-offs, received supplemental barriers to care information, and care reengagement support.
**Bridging Factors**	Community and academic partnerships	- Worked with YSM staff throughout the study period to implement the intervention and address any issues highlighted by the DIS or clinic staff.		- The University of Pennsylvania Center for AIDS Research (CFAR) served as the Community Advisory Board for the project.

#### Interorganizational networks

Throughout the RCT, CoRECT site teams continued to work closely with partnering clinics to maintain buy-in and address any challenges or concerns such as staffing changes within the clinic or CoRECT team.

#### Participant recruitment

The sites and the participating clinics used case conferences to review and discuss the eligibility of each potential OOC case. These conferences generally entailed DPH CoRECT team members (e.g., data managers, project coordinators, or epidemiologists) meeting with individual clinic points of contact, typically case managers, to discuss every patient identified by the reconciliation process to be OOC with the goal of finalizing a list of eligible patients for randomization. Clinicians, social workers, and nursing staff were also invited to participate to provide additional case-specific information. CTDPH/YSM had multiple participating clinics which necessitated the adoption of quarterly case conferences. MDPH allowed clinics to submit either “all patients” or OOC lists via three methods based on their capacity and individual preference. PDPH continued with the procedures that they developed during their pilot study (see Table 1; Figure 1).

As for the case conferences, PDPH developed a structured procedure led by the D2C project coordinator, primarily in-person. However, when the list of cases to review were less than five, meetings could be held over the phone. MDPH developed a semi-structured procedure led by the MDPH D2C epidemiologist, while CTDPH/YSM took a more unstructured approach led by the YSM CoRECT data manager (Table 1). All three sites experienced instances that required a second case conference to complete the review of potential OOC cases due to high OOC caseloads and the time-intensive conferencing process.

#### Fidelity monitoring/support

During the RCT, each site held monthly internal meetings and periodic project status meetings with clinical partners to provide support and discuss concerns and challenges. In retrospect, CTDPH/YSM did not hold regular monitoring meetings with local health departments that employed the DIS, which eventually posed a limitation. MDPH conducted routine reviews of field staff activities associated with HIV care re-engagement activities and held monthly meetings with field staff to discuss cases and new barriers/challenges.

#### Staffing

A common challenge faced was staff turnover throughout the course of the project among CoRECT teams, field staff, and partnering clinics which affected the productivity and efficiency of case conferences and patient follow-up. New team members or field staff required updated training to familiarize themselves with the project protocols and processes as well as to establish and build strong working relationships with the multiple different collaborators.

#### Innovation characteristics

CoRECT’s use of both surveillance and clinic data to identify truly OOC patients required ongoing flexibility to adjust procedures consistent with clinic capacity and operational features related to case identification and DIS field activities.

### Sustainment phase

Sites encountered challenges such as changes in leadership at health departments and participating clinics, health department reorganization, and funding after the trial ended, which impacted determining what components of the CoRECT study activities that could be sustained (Table 3).

**Table 3:** Sustainment phase CoRECT project.

	EPIS constructs	Connecticut DPH	Massachusetts DPH	Philadephia DPH
**Outer context**	Sociopolitical	- National focus to sustain care engagement and effective intervention activities for those out-of-care (OOC).	- National focus to sustain care engagement and effective OOC intervention activities for those OOC.	- National focus to sustain care engagement and effective OOC intervention activities for those OOC.
	Funding	- Need to examine how best to continue intervention or revised version without additional CDC funding. - Seeking future Data to Care (D2C) funding opportunities.	- By leveraging an integrated approach to HIV/STI follow-up and other resources, a scaled down set of activities have been continued without the additional CDC funding. - Evaluate which intervention components were the most successful and could be most effectively integrated into standard of care.	- By leveraging other funding sources, similar activities have been continued without the additional CDC funding. - Evaluated which intervention components were the most successful and could be most effectively integrated into standard of care.
	Interorganizational networks	- DIS dedicated to OOC work need to be integrated into clinics’ workflow.	- Establish identified intervention components as standard of care. - DPH continues supporting educational and joint programs with providers and community organizations.	- Established identified intervention components as standard of care. - Expanded activities to providers/clinics that were not part of the RCT.
	Leadership	- Priority given to apply data to public health action and promoting equity in access to healthcare and achieving health outcomes. - Organizational restructuring impaired the continuation of the CoRECT intervention.	- Priority given to apply data to public health action and promoting equity in access to healthcare and achieving health outcomes. - Administrative buy-in and engagement of clinical leads/managers is needed to facilitate sustainability. - Invest in capacity for public health intervention and infrastructure for data-to-care strategy.	- Administrative buy-in and engagement of clinical leads/managers is needed to facilitate sustainability. - Priority given to apply data to public health action and promoting equity in access to healthcare and achieving health outcomes.
**Inner context**	Organizational characteristics	- Work with clinics to re-engage OOC HIV patients. - Reorganization plans include creation of new positions, such as D2C Coordinator. - Future interventions will need to address heterogeneity in the DIS workforce.	- Support generation of OOC patient lists. - Provide capacity building assistance to clinics to identify OOC patients and improve linkage to care. - Work with clinics to re-engage OOC HIV patients. - Collaborate with clinics to deploy field staff to support patient (re)engagement.	- Work with clinics to re-engage OOC HIV patients. - A HIV specific Field Services Program was developed. - The HIV specific Field Services Program and clinics work together to re-engage HIV patients into care, using D2C models (including CoRECT) that incorporated ARTAS and patient navigation.
	Individual adopter characteristics	- Follow up D2C plan is being reformulated.	- D2C activities ongoing, with variability across participating clinics; ongoing evaluation of D2C strategies to optimize effectiveness and efficiency.	- D2C activities are ongoing and being scaled up. - Re-engagement activities through the Field Services Program are required for Ryan White funded providers.
**Inner context**	Leadership	- New leadership and reorganization of branches.	- Stable leadership, and continued support for integrated approach to field services; stable organizational management of D2C and field services.	- Stable leadership, and continued support for field services. - Stable organizational management of D2C and field services.
	Participant recruitment	- Assessing ways to implement similar procedures. - No current protocol for generating OOC lists to share with clinics.	- Assessing ways to implement similar procedures and apply new definitions to out-of-care, engagement, and retention.	- Continued to conduct case conferences. - Virtual meetings are held and sites also conduct independent case conferences with periodic review.
	Fidelity monitoring/support	- Hired D2C Coordinator.	- D2C and re-engagement have been integrated into field activities; use of routine CQI.	- Fidelity to the intervention by Field Services Staff is monitored and supported by the Field Services Program Lead and Supervisor.
	Staffing	- Use of newly hired DIS impacts sustainability. - Reviewing reorganized DIS roles and responsibilities such as HIV OOC work. - Staffing changes need to be considered.	- Integration of key components into existing workflows and staff functions increases the intervention sustainability.	- Field Services Program improved sustainability because it resolved issues of competing staff priorities.
**Innovation**	Innovation characteristics	- Provided clinics capacity-building assistance.	- Maintained infrastructure and processes for data sharing with clinics. - Some clinics still use data to identify OOC patients, with variability in process for use, and frequency for generating OOC lists. - Maintained integration of HIV/STD field staff. - Maintained capacity for re-engagement follow-up. - Provided clinics capacity building assistance to maintain D2C practices; extended lessons learned to other infections, notably hepatitis C.	- Maintained infrastructure and processes for data sharing with clinics. - Provided clinics capacity-building assistance. - All clinics still operating in Philadelphia still use data to identify OOC patients. - Maintained capacity for re-engagement follow-up.
**Bridging factors**	Community and academic partnerships	- Future assessments are needed to examine the incorporation of this collaboration in future activities. - YSM has analyzed CoRECT sites for *post hoc* analyses and submitted manuscripts. - YSM has harnessed additional grants to foster this collaboration	- Consulted with participating clinics to obtain feedback regarding strategies to optimize OOC definitions and processes. - Consulted with advisory committees, including consumers, to provide input and support.	- The University of Pennsylvania Center for AIDS Research (CFAR) served as the Community Advisory Board for the project until the COVID-19 pandemic. - Consulted with participating clinics to obtain feedback regarding strategies to optimize OOC definitions and processes.

#### Leadership

While all sites endorsed general principles of D2C, namely, continuing to actively advocate for HIV re-engagement activities using combined public health and clinical data, leadership challenges were notable. CTDPH faced the dual challenges of external study leadership and organizational restructuring with the hiring of new leaders. PDPH expanded its leadership by transitioning their D2C project coordinator to the head of the HIV specific Field Services Program. In contrast, there were no major changes in MDPH’s leadership or structure.

#### Organizational characteristics

PDPH maintained similar case identification and patient follow-up activities and retained their dedicated field staff through the reallocation of resources and the development of the HIV-specific Field Services Program, which took the lead in managing the field staff responsible for OOC follow-up activities. Additionally, personnel responsible for data analysis and management were grouped together in the HIV Surveillance Unit. The creation of this program has allowed for the expansion of D2C activities based on CoRECT to all Ryan-White funded facilities. In MDPH, the integration of CoRECT activities into the routine workflow of HIV/STI field epidemiologists and surveillance epidemiologists allowed for the continuation of patient follow-up activities, although in a more limited capacity. In contrast, without YSM to provide project management support and resources to support dedicated field staff, CTDPH had to halt their CoRECT follow-up activities. CTDPH has since hired a D2C coordinator and continues to re-visit their approach.

#### Participant recruitment

All three trial sites highlighted the need to include procedures to update OOC definitions based on application of evolving epidemiology and updated clinical guidelines and practices. Since CoRECT concluded, there were also the changes in health care delivery and access related to the COVID-19 pandemic that will certainly have impacts on how we ascertain whether someone is truly OOC and might need assistance.

#### Staffing

Staffing significantly impacts program sustainability, through turnover, maintenance of institutional knowledge and relationships, leadership, or existing organizational structure. PDPH and MDPH retained/sustained the field, surveillance, and D2C data management positions involved in CoRECT. Furthermore, PDPH was able to expand the number of dedicated D2C staff through the Field Services Program with new funding from the state of Pennsylvania. CTDPH were unable to retain their CoRECT-specific field staff and YSM project management staff; however, as part of an ongoing reorganization plan, they have included new internal positions such as a Data to Care Coordinator.

#### Community and academic partnerships

Even with the expertise and other resources offered by an external academic partner for project management, such as with CTDPH, long-term sustainability due to sub-optimal infrastructure development is challenging. However, one advantage of having an academic partner is the ability to create post-hoc analyses and publish in peer-reviewed journals (23). Outside of academic partners, other community relationships were also important in the sustainment of this work. MDPH and PDPH recognized that consulting with collaborating clinics, as well as advisory committees, including consumers, could provide beneficial input and support for future D2C interventions. PDPH continued their partnership with the University of Pennsylvania CFAR until the start of the COVID-19 pandemic.

## Discussion

To our knowledge, CoRECT was the first prospective RCT to implement and assess the use of a D2C strategy accompanied by an intervention component for re-engaging newly OOC PWH (10, 11, 23). While the study demonstrated successful initial re-engagement in care for OOC persons compared to the SOC and the Philadelphia site also demonstrated successful retention in care at 12 months, the specific implementation components have not been fully analyzed (10). We used the EPIS framework to operationalize a systematic approach to detail the implementation processes of the three CoRECT sites to highlight the differences in jurisdictional approaches to data sharing, case reconciliation, patient follow-up activities, and sustainability. The consolidation of this information identified useful lessons learned for future D2C activities as well as dissemination to other jurisdictions.

Utilization of the EPIS framework retrospectively was well-suited for this assessment given its emphasis on implementation, as well as initial planning and long-term sustainability. EPIS allowed us to explore pre-defined contextual factors across both jurisdiction and stage and enabled a more nuanced understanding of the complex interplay between the intervention, the stakeholders involved, and larger socio-ecological considerations. This provided valuable insights into challenges and facilitators as well as future D2C delivery and sustainability.

Each trial site approached CoRECT grounded in their own context and informed by a multitude of factors including prior D2C experience, data management infrastructure, staffing capacity and roles relative to HIV patient recruitment navigation, and relationships with healthcare clinic partners. While CoRECT was a research study funded by the CDC, retrospective analysis using the EPIS framework, including the preparation and implementation components, highlighted the critical role of “ownership” of the D2C initiative by the health departments from the standpoint of leadership and organizational infrastructure. The approaches taken by PDPH and MDPH emphasized internal investment by using DPH HIV surveillance staff, building new processes to support OOC re-engagement, and incorporating OOC re-engagement activities of field staff into the existing health department organizational structure and processes. Although PDPH had prior D2C experience with a data exchange process, the pilot study proved to be extremely helpful in soliciting feedback from their CoRECT team and clinical collaborators to craft the full-scale field intervention and fine-tune their D2C process. This initial prioritization in the exploratory and preparation phases facilitated the development of a more sustainable intervention, which was further supported by the creation of an intraorganizational HIV-specific Field Services Program and leveraging other funding sources. MDPH incorporated lessons learned from prior D2C experience and through experience with routine HIV patient navigation to leverage existing infrastructure for data sharing and incorporated case assignments into existing field staff’s workflow. This sustainability focused approach allowed MDPH to continue some aspects of the CoRECT intervention after conclusion of the CoRECT study.

In contrast, CTDPH lacked prior D2C experience but prior research efforts and a commitment to principles inherent in D2C led them to partner with an academic institution, YSM. Newly created processes and infrastructure including hiring of dedicated CoRECT field staff through local health departments aided in the successful implementation of CoRECT, however, they negatively impacted sustainability because the YSM and field staff were outside of the CTDPH structure. Fortunately, the CTDPH was able to identify ways to address future D2C activities including the use of pilot testing, centralizing DIS activities within the CTDPH, designating a D2C coordinator, and including field and healthcare clinic staff in discussion regarding long-term feasibility.

A fundamental principle that emerged was adaptability, specifically the importance of being able to modify the procedures because there is no one-size-fits-all approach to designing/implementing a D2C project given the variability in target populations, health department characteristics/structure, and participating clinic characteristics. This was particularly critical given the changing clinical standards of care, evolving public health approaches such as integrated data systems, and updated goals of the National HIV/AIDS Strategy and Ending the HIV Epidemic initiatives (1). Adaptability was also indispensable given the need for flexibility and need for local tailoring among key participants at national and local levels. The successful strengthening of collaboration between health departments and healthcare clinics, as an explicit goal of CoRECT, required negotiation and compromise in building interorganizational networks.

Challenges identified during this assessment, including those related to leadership, staffing and turnover; evolving clinical practices regarding OOC definitions; sustainability of community and academic partnerships, fidelity monitoring and data management discrepancies, and inconsistent buy-in, underscore the importance of evaluation and integration steps throughout the process. While many of these challenges were addressed through prioritization of establishing standardized protocols, streamlined communication, consistent training practices, and clear expectations, flexible strategies that can accommodate changes within public health and clinical settings remain paramount for continued D2C evolution as it is refined in new contexts.

This retrospective assessment not only provides an overview of each trial site’s approach to the CoRECT intervention and the implementation process, but also provides some key take-aways, which may prove useful for those interested in developing and implementing their own D2C activities. We would like to highlight the following key principles as a guidance for health departments considering implementing or enhancing similar activities.

- Tailor D2C structures and activities to the jurisdictional needs based on their unique characteristics in order to be effective across varying contexts.
- Develop appropriate and sufficient technical and staffing infrastructure to enable the use of multiple different sources of data to supplement surveillance data (e.g., patient clinical records, Medicaid claims, medical case management data, or Ryan White program data) even as integrated data platforms continue to be explored.
- Appoint a health department “champion” for D2C who will serve as the internal point person for health department staff and clinic personnel. Ideally, the “champion” will have extensive HIV experience and existing relationships with the clinics, including knowledge of their patient population and data.
- Create follow-up and outreach activities by health department personnel that are feasible and sustainable within the jurisdiction’s capacity, organization, and infrastructure.
- Include stakeholders representing core functions/components (e.g., Health Department Medical Directors, Health Department HIV Directors, disease surveillance staff, field staff, program managers or administrators, clinic administrators, continuous quality improvement staff, patient navigators, and clinic data management) in the early stages of the intervention design process. This could entail the implementation of a pilot study to allow for feedback to address challenges prior to initiating the full intervention.
- Create ongoing and regular opportunities, such as project status meetings, for health department and clinic staff to provide feedback regarding barriers/challenges that have been identified. These meetings can also serve to maintain good intra- and interorganizational communications and serve as learning collaboratives.
- Build flexibility into all components of the activities, including, but not limited to the case reconciliation process (e.g., case conference location and methodologies for providing patient lists), OOC definitions, and have the flexibility to align/respond to organizational capacity, infrastructure, and operational efficiencies.
- Develop and incorporate the administration of D2C data sharing/management and follow-up activities within the existing health department organizational structure. This may include establishing and supporting cross department/division engagement (e.g., HIV surveillance staff working closely with STI field staff or IT department).
- Use a structured format for case conferences to review cases with clinic staff in a timely and efficient manner.
- Develop and document written procedures and fidelity processes early in the implementation process to ensure consistent implementation and accommodate for changes in health department and clinic staff and organization. Apply standardized procedures that can be periodically revisited to ensure they remain maximally responsive to environmental and contextual factors, so interruption in activities can be kept to a minimum.
- Monitor changes in guideline implementation, clinical practices, and standards of care to make timely updates to D2C OOC case identification algorithms and follow-up activities as necessary.
- Organize regular training and educational opportunities to sustain the D2C activities for health department and clinic staff.

### Limitations

A limitation of this evaluation was the inability to obtain comprehensive feedback on the implementation process from field and clinic staff due to staff turnover. Future evaluation projects could address this by collecting feedback prospectively during the four EPIS framework stages. There were several strengths including a comprehensive appraisal of comparing the implementation processes across three CoRECT sites in a structured format and generate lessons learned for future D2C activities. By combining all three sites into a single evaluation, lessons learned are more robust, nuanced, and useful for future implementation.

## Conclusion

In conclusion, we found that while the CoRECT study framework successfully provided a general blueprint for health departments to implement a D2C intervention for improving re-engagement in HIV care, it is important for jurisdictions to customize it based on their own needs and evolving practices. The lessons learned in the exploratory, preparation, implementation, and sustainment phases of implementation by CTDPH/YSM, PDPH, and MDPH provide initial starting points and critical aspects that are critical for leadership to consider when implementing D2C.

## Data availability statement

The original contributions presented in the study are included in the article/Supplementary material, further inquiries can be directed to the corresponding author.

## Author contributions

HE led the review and the writing processes. SGL assisted with major draft revisions and provided site specific information. BJ, KR, BG, and MB provided site specific information and assisted in the editing process. MV, KAB, AD, and LR contributed to the concept, site specific information. FLA provided guidance in the use of implementation frameworks. All authors contributed to the article and approved the submitted version.
