# Supplementary material for: Using the exploration, preparation, implementation, sustainment (EPIS) framework to assess the cooperative re-engagement controlled trial (CoRECT)
Source: Front Public Health. 2023 Dec 1;11:1223149. doi: 10.3389/fpubh.2023.1223149 (PMC10722986; doi:10.3389/fpubh.2023.1223149)
Supplement: Supplementary file 1 [file Table_1.DOCX]

Supplemental Table 1. Exploration Phase CoRECT Project

|  | **EPIS CONSTRUCTS** | **CONNECTICUT DPH** | **MASSACHUSETTS DPH** | **PHILADEPHIA DPH** |
| --- | --- | --- | --- | --- |
| **OUTER CONTEXT** | **Sociopolitical** | - National priority.  - CDC prioritized “data-to-care” (D2C) for HIV interventions.  - Prioritized HIV care retention and learning more about HIV care disengagement.  - Interested in deployment of public health workforce and developing infrastructure to optimize D2C. | - National priority.  - CDC prioritized D2C for HIV interventions.  - Interested in and prior experience implementing D2C strategies for facilitating engagement and care retention.  - Interested in and prior experience deploying public health workforce and developing infrastructure to optimize D2C.  - Interested in understanding patient characteristics and factors associated with HIV care disengagement. | - National priority.  - CDC prioritized D2C for HIV interventions.  - Interested in and prior experience implementing D2C strategies for facilitating engagement and care retention.  - Prioritized HIV care retention and learning more about HIV care disengagement.  - Interested patient characteristics and factors associated with HIV care disengagement.  - Interested in deployment of public health workforce and developing infrastructure to optimize D2C. |
|  | **Funding** | - DPH leadership pursued CoRECT funding.  - CDC announced funding for three trial sites.  - Clinics could leverage other support for HIV management including state and federal funding for HIV medical case management and care coordination. | - DPH leadership pursued CoRECT funding.  - CDC announced funding for three trial sites.  - Clinics could leverage other support for HIV management including state and federal funding for HIV medical management and care coordination. | - DPH leadership pursued CoRECT funding.  - CDC announced funding for three trial sites.  - Clinics and DIS could leverage other support for HIV management including state and federal funding for HIV medical management and care coordination. |
|  | **Interorganizational Networks** | - Looks for new ways to establish and strengthen relationships with clinics and academic partners.  - Participates in HIV meetings with external partners. | - Continually looks for new ways to establish and strengthen relationships with clinics and HIV care providers throughout the state.  - Existing collaboration with variety of clinical entities to implement, HIV services, and research and evaluation activities, including D2C strategies. | - Continually looks for new ways to establish and strengthen relationships with clinics.  - Existing collaboration with variety of clinical entities to implement, HIV services, and research and evaluation activities, including D2C strategies. |
|  | **Leadership** | - History collaborating with Yale School of Medicine (YSM) on grant projects.  - Prioritized data to public health action, and to develop/adopt strategies for quality improvement.  - Prioritized the promotion of equity in access to healthcare and achieving health outcomes. | - Prioritized applying data to public health action and developing/adopting strategies to promote and support continuous quality improvement.  - Prioritized promoting equity in access to healthcare and achieving health outcomes. | - Engaged and passionate.  - Prioritized applying data to public health action and developing/adopting strategies to promote and support continuous quality improvement.  - Prioritized promoting equity in access to healthcare and achieving health outcomes. |

Supplemental Table 1. Exploration Phase CoRECT Project (continued)

|  | **EPIS CONSTRUCTS** | **CONNECTICUT DPH** | **MASSACHUSETTS DPH** | **PHILADEPHIA DPH** |
| --- | --- | --- | --- | --- |
| I**NNER CONTEXT** | **Organizational characteristics** | - Existing experience and infrastructure to implement additional HIV follow-up to newly diagnosed.  - Has strong experienced HIV surveillance staff with ability to generate HIV care continuum estimates. | - Prior experience with data-to-care strategies.  - Had prior needs assessments experience.  - Had prior deployment of public health staff to facilitate engagement in HIV medical care. | - Prior experience with data-to-care strategies.  - Infrastructure to implement additional HIV follow-up.  - Planned the use of pilot study to supplement prior D2C experience. |
|  | **Leadership** | - Seeks out new opportunities to expand ability to assist patients and healthcare providers. | - Seeks out new opportunities to expand ability to assist patients and healthcare providers. | - Seeks out new opportunities to expand ability to assist patients and healthcare providers. |
|  | **Participant Recruitment** |  | - Interested in new definitions of out-of-care, and engagement and retention in care strategies. |  |
|  | **Staffing** | - Existing staff were not positioned to take on OOC DIS activities.  - Planned to hire CoRECT specific DIS. | - Integrated out-of-care engagement into workflow of staff, including both surveillance and field epidemiologists.  - Planned to use existing field staff, epidemiologists. | - Planned to hire local DIS. |
| **INNOVATION** | **Innovation characteristics** | - Tailored case identification process and intervention characteristics to meet DPH and participating clinics’ needs.  - Modified Anti-Retroviral Treatment and Access to Services focused on short-term engagement. | - Tailored case identification process and intervention characteristics to align with DPH and collaborating clinic capacity and operations.  - Short-term assistance with the possibility of longer-term assistance and motivational counseling. | - Tailored case identification process and intervention characteristics to meet DPH and participating clinics’ needs.  - Modified Anti-Retroviral Treatment and Access to Services focused on long-term engagement. |
| **BRIDGING FACTORS** | **Community and academic partnerships** | - Partnered with YSM. |  |  |

Supplementary Table 2. Preparation Phase CoRECT Project

|  | **EPIS CONSTRUCTS** | **CONNECTICUT DPH** | **MASSACHUSETTS DPH** | **PHILADEPHIA DPH** |
| --- | --- | --- | --- | --- |
| **OUTER CONTEXT** | **Sociopolitical** | - A focus to continue establishing and strengthening relationships with Yale School of Medicine (YSM), healthcare facilities, and individual providers. | - A focus to continue strengthening relationships with healthcare facilities and individual providers.  - Enhance collaborations with healthcare facilities to share data for public health action. | - A focus to continue establishing and strengthening relationships with healthcare facilities, and individual providers.  - Enhance collaborations with healthcare facilities to share data for public health action. |
|  | **Funding** | - Prior CDC Surveillance grant funding supported traditional DIS positions.  - New funding supported CoRECT specific DIS  - No funding was provided to clinics. | - Funding allowed provision of financial support to the participating health clinics to support project activities including running OOC lists, case conference participation, data management, and local project administration.  - Funding provided partial support for dedicated D2C epidemiologist. | - New funding supported new local DIS.  - No funding was provided to clinics. |
|  | **Interorganizational Networks** | - Leadership and YSM visited key stakeholders to secure buy-in by leveraging existing relationships.  - Data sharing agreements between clinic sites and DPH was established within the context of IRB. | - Existing advisory committees and consortia, as well as established clinical contacts with publicly funded and non-funded clinics. | - Leadership and DPH staff visited key stakeholders in clinics to secure buy-in by leveraging existing relationships.  - Data sharing agreements between clinic sites and DPH were established. |
|  | **Leadership** | - DPH and clinic leadership were interested/engaged in the intervention and implementation process. | - DPH and clinic leadership were interested/engaged in the intervention and implementation process. | - DPH and clinic leadership were interested/engaged in the intervention and implementation process. |
| **INNER CONTEXT** | **Organizational characteristics** | - Extant staff and YSM provided management and oversight.  - New DIS housed in local health departments.  - Clinics used the study’s scope to identify staff and processes needed to execute these activities.  - Developed data collection tools. | - Extant DPH staff provided management and oversight.  - Identified staff responsible for project components including data-to-care epidemiologist.  - Clinics used the study’s scope to identify staff and processes needed to execute project activities.  - Developed data collection tools. | - Extant staff provided management and oversight.  - Identified staff responsible for project components including data-to-care data staff.  - Clinics used information on the study’s scope to identify staff and processes needed to execute these activities.  - Developed data collection tools. |

Supplementary Table 2. Preparation Phase CoRECT Project (continued)

|  | **EPIS CONSTRUCTS** | **CONNECTICUT DPH** | **MASSACHUSETTS DPH** | **PHILADEPHIA DPH** |
| --- | --- | --- | --- | --- |
| **INNER CONTEXT** | **Individual adopter characteristics** | - YSM assisted in the development of a successful and sustainable care re-engagement intervention.  - Clinic partners facilitated institutional buy-in for practice change necessary for study and collaboration.  - **Clinic partners and CoRECT specific field staff did not interact directly with CTDPH staff.** | - Clinic partners facilitated institutional buy-in for practice change necessary for study and collaboration.  - Able to incorporate lessons learned from prior D2C projects.  - Planned integration of randomized case assignments into standard workflow structure. | - Clinic partners facilitated institutional buy-in for practice change necessary for study and collaboration.  - Able to incorporate lessons learned from prior D2C projects.  - Use of a pilot study to assess initial protocols and follow-up activities.  - Able to modify case identification protocol based on pilot study findings. |
|  | **Leadership** | - Primary investigators were engaged and supportive.  - YSM well-versed in implementation science.  - YSM lead ARTAS framework revision and trainings. | - Primary investigators were engaged and supportive. | - Primary investigators were engaged and supportive. |
|  | **Participant Recruitment** | - Planned quarterly electronic data transmission.  - Planned quarterly conference call with clinics.  - During calls, potential cases were reviewed to determine final eligibility status. | - Planned monthly electronic data transmission.  - Planned monthly conference call with clinics.  - During calls, potential cases were reviewed individually to determine final eligibility status. | - Planned monthly electronic data transmission.  - Planned monthly in-person conferences with clinics.  - During meetings, potential cases were reviewed individually to determine final eligibility status. |
|  | **Fidelity monitoring/support** | - Not well defined. | - Variables and monitoring features were added to established surveillance database for project evaluation and quality assurance. | - D2C Project Coordinator conducted monitoring activities with DIS staff to assure fidelity to the ARTAS model.  - Outcomes in eHARS routinely monitored by leadership. |
|  | **Staffing** | - DIS, YSM, DPH epidemiologists and clinic staff received additional training including modified ARTAS and case identification.  - Enhanced technical infrastructure to facilitate and streamline data to care strategy. | - Field staff, DPH epidemiologists, and clinical staff received additional training on the study protocol including follow-up procedures and case identification.  - Enhanced technical infrastructure to facilitate and streamline data to care strategy. | - DIS, DPH epidemiologists and clinic staff received additional training including modified ARTAS, the study protocol, and case identification.  - Enhanced technical infrastructure to facilitate and streamline data to care strategy. |

Supplementary Table 2. Preparation Phase CoRECT Project (continued)

|  | **EPIS CONSTRUCTS** | **CONNECTICUT DPH** | **MASSACHUSETTS DPH** | **PHILADEPHIA DPH** |
| --- | --- | --- | --- | --- |
| **INNOVATION** | **Innovation characteristics** | - Developed infrastructure and strategies using both clinic and surveillance data to identify OOC.  - Prioritized full integration of HIV and STD activities.  - Collaborate with clinics to facilitate reengagement. | - Developed infrastructure and strategies using both clinic and surveillance data to identify OOC.  - Prioritized full integration of HIV and STD activities.  - Collaborate with clinics to facilitate reengagement. | - Developed infrastructure and strategies using both clinic and surveillance data to identify OOC.  - Prioritized full integration of HIV and STD activities.  Collaborate with clinics to facilitate reengagement. |
| **BRIDGING FACTORS** | **Community and academic partnerships** | - Collaborated with YSM in the development of the intervention.  - YSM oversaw randomization, data management, and case conferences. |  | - The University of Pennsylvania Center for AIDS Research (CFAR) served as the Community Advisory Board for the project. |
